# Modeling and forecasting age-specific drug overdose mortality in the United States

**DOI:** 10.1140/epjs/s11734-023-00801-z

**Published:** 2023-04-26

**Authors:** Lucas Böttcher, Tom Chou, Maria R. D’Orsogna

**Affiliations:** 1 Department of Computational Science and Philosophy, Frankfurt School of Finance and Management, 60322 Frankfurt am Main, Germany; 2 Department of Computational Medicine, University of California, Los Angeles, Los Angeles, 90095 CA USA; 3 Department of Mathematics, University of California, Los Angeles, Los Angeles, 90095 CA USA; 4 Department of Mathematics, California State University at Northridge, Los Angeles, 91330 CA USA

## Abstract

Drug overdose deaths continue to increase in the United States for all major drug categories. Over the past two decades the total number of overdose fatalities has increased more than fivefold; since 2013 the surge in overdose rates is primarily driven by fentanyl and methamphetamines. Different drug categories and factors such as age, gender, and ethnicity are associated with different overdose mortality characteristics that may also change in time. For example, the average age at death from a drug overdose has decreased from 1940 to 1990 while the overall mortality rate has steadily increased. To provide insight into the population-level dynamics of drug overdose mortality, we develop an age-structured model for drug addiction. Using an augmented ensemble Kalman filter (EnKF), we show through a simple example how our model can be combined with synthetic observation data to estimate mortality rate and an age-distribution parameter. Finally, we use an EnKF to combine our model with observation data on overdose fatalities in the United States from 1999 to 2020 to forecast the evolution of overdose trends and estimate model parameters.

## Introduction

The number of drug overdose fatalities in the United States has been steadily increasing over the past 20 years [1, 2]. Between 1999 and 2020, more than 900,000 drug overdose deaths were reported in the United States. In 2020 alone, almost 100,000 people died from injury or poisoning from drugs of abuse (mainly opioids and psychostimulants), constituting a 32% rise over 2019. According to provisional mortality data [3], this trend has continued throughout 2021.

A study [1] that examined the exponential growth in drug overdose deaths between 1979 and 2016 in the United States reveals that the drug types causing these rises have changed over time. During the 1980s and 1990s, the majority of fatal drug overdoses were due to illegal substances such as heroin and cocaine. Successive overdose waves were driven by prescription opioids in the 2000s, followed briefly by heroin in 2010, and, beginning in 2013, by synthetic opioids. The synthetic opioid wave persists to this day, as the majority of US overdose deaths are due to fentanyl and its derivatives. There is also substantial variability in the demographic patterns of drug overdose deaths. While cocaine and prescription drugs mostly led to increased mortality among 40- to 50-year-olds, current fentanyl use is accompanied by fatality rate increases among 20- to 40-year-olds. In addition to age, factors such as gender, race, and place of residence are also associated with variations in drug overdose risk [4].

The majority of studies analyzing the spatiotemporal evolution of overdose mortality are mainly descriptive and rely on data visualization and statistical analysis of past data. In this work, we instead use an age-structured model [5, 6] to mechanistically study the drug epidemic in the United States. The model is then used in conjunction with empirical data to forecast the short-term evolution of overdose mortality through an ensemble Kalman filter (EnKF), a data assimilation technique [7–9].

Age-structured models (also known as Kermack–McKendrick models) can be used to mathematically describe the evolution of distinct population categories (e.g., susceptible and dead), where the dynamics and interactions among categories may depend on the distribution of age in the population. Different variants of age-structured models have been developed and applied to model heroin addiction as an epidemic [10–17]. Such models have also been applied to mechanistically describe cellular processes [18, 19] and population dynamics associated with social interactions [20], birth control policies [21], and COVID-19 mortality [22–24].

The EnKF, which we use to combine observation data with an age-structured model of overdose mortality, originated from research activities in the geophysical sciences and has found various applications in problems that require combining high-dimensional dynamical systems with observation data [25]. Kalman filtering and related data assimilation methods (e.g., Bayesian Markov chain Monte Carlo) have been used in computational biology and medicine to estimate model parameters [26–29], identify patients with antibiotic-resistant bacteria in hospital wards [30], and develop risk-dependent contact interventions in epidemic management [31]. Within computational social science, data assimilation methods have proven useful in combining mechanistic models with survey data, e.g., to study the evolution of political polarization in the United States [32].

In Sect. 2 we present a general age-structured model that includes age-dependent addiction and age-specific mortality. We also discuss approximations that admit analytical solutions. The basic concepts underlying the EnKF are outlined in Sect. 3. In Sect. 4 we adapt our general age-structured model to describe a population suffering from substance use disorder (SUD). We then describe the available drug overdose data and illustrate how the EnKF is applied to our model and dataset. Finally, we conclude our work with a discussion and future outlook in Sect. 5.

## A general age-structured mortality model

Our starting point is the general age-structured model

1 $$\begin{aligned} \left[ \frac{\partial }{\partial a} + \frac{\partial }{\partial t} \right] n (a, t)&= - \mu (a, t) n (a, t)+ p(a,t),  \end{aligned}$$

where $n(a,t) \textrm{d}a$ is the number of individuals (i.e., people with SUD in our application) with age between *a* and $a + \textrm{d}a$ at time *t*. We assume this population dies at rate $\mu (a,t)$, and that there is an influx rate *p*(*a*,*t*). The initial conditions at $t=t_0$ and $a=0$ are specified via $n(a, t=t_0) = \rho (a)$ and $n(a=0, t) = g(t)$, where $\rho$ and *g* are non-negative functions such that $g(t \rightarrow t_0) = \rho (a \rightarrow 0)$. We specifically set $g(t) = 0$, implying that no population of age $a=0$ exists at any time. In the context of modeling overdose mortality, this means that the number of addicted newborns is assumed to be zero. Note that this model is different from the original McKendrick model [5], in which an age-dependent birth rate generates newborns through a self-consistent boundary condition on *n*(*a*,*t*). To solve Eq. (1) analytically, we use the method of characteristics and distinguish the two cases $a \ge t - t_0$ and $a < t - t_0$. For $a \ge t-t_0$, the characteristic will begin at $t=t_0$ and *n*(*a*,*t*) will remain constant along $a = t -t_0$. When $a < t - t_0$ the characteristic will begin at $a = 0$ and *n*(*a*,*t*) will remain constant along $t = a + t_0$. The formal solution to Eq. (1) can then be expressed as

$$\begin{aligned}   n(a, t)  =\left\{ \begin{array}{lll} & \rho (a -t + t_0) e^{-\int _{t_0}^t \mu (a-t+s, s)\, \textrm{d}s} + \int _{t_0}^{t} p(s + a - t, s) e^{-\int _{s}^t \mu (a-t+s', s') \textrm{d}s'} \textrm{d}s \qquad\qquad&{} (a \ge t - t_0)\,\, \, \qquad\qquad \qquad\,\, (2) \\\\\quad & \int _{0}^{a}\! p(s, s +t -a ) e^{-\int _{s}^a \mu (s', s' + t - a)\, \textrm{d}s'} \textrm{d}s \qquad\qquad&{} (a < t - t_0). \,\,\qquad\qquad \qquad\,\, (3) \end{array}\right. \end{aligned}$$

As a specific example we set the initial time $t_0 = 0$, fix the initial condition $\rho (a) = 0$, and impose a constant death rate $\mu (a,t) = \mu$. We further assume an influx rate $p(a,t) = p(a) = a e^{-\lambda a}$ which has a maximum at age $\lambda ^{-1}>0$. Equations (2) and (3) become

$$\begin{aligned}   {n(a, t) } =\left\{ \begin{array}{lll} {e^{-\lambda (a-t)} \over (\lambda - \mu )^2} \Big [e^{-\mu t }\big (1 + (a-t)(\lambda - \mu ) \big )- \big (1+ a(\lambda - \mu )\big ) e^{-\lambda t}\Big ] \qquad\qquad&{} (a \ge t )\,\,\quad\qquad\qquad\qquad\qquad\quad\qquad\qquad\quad \,\, (4) \\ \frac{1}{(\lambda - \mu )^2}\Big [e^{-\mu a} -\big (1 + a (\lambda - \mu )\big ) e^{-\lambda a}\Big ] \qquad\qquad&{} (a < t).\quad\,\,\quad\qquad\qquad\qquad\qquad\quad\qquad\qquad  (5) \end{array}\right. \end{aligned}$$

The function $p(a) = a e^{-\lambda a}$ describes an influx of people of mean age $2\lambda ^{-1}$ that suffer from an SUD. Using this functional form, the number of SUD cases that are much younger/older than $2\lambda ^{-1}$ is small compared to the number of SUD cases with an age of about $2\lambda ^{-1}$. The distribution of overdose cases in the US population follows a qualitatively similar trend [33]. We use this analytically tractable example in Sect. 3 to explain how age-structured models of the form presented in Eq. (1) can be combined with Kalman filters to learn model parameters from noisy observations. In Sect. 4, we describe *p*(*a*) by a more general linear combination of two gamma distributions to connect our model of drug overdose mortality with corresponding data from the Centers for Disease Control and Prevention (CDC) Wide-ranging Online Data for Epidemiologic Research (WONDER) database.

Observe that *n*(*a*,*t*) in Eqs. (4) and (5) is continuous for $a=t$ and that the maxima of Eq. (4) are located along the trajectory

6 $$\begin{aligned} \begin{aligned} a_{\textrm{max}} (t) = \frac{t}{1 - e^{-(\lambda - \mu ) t}} - \frac{\mu }{\lambda (\lambda - \mu )}, \end{aligned} \end{aligned}$$

where $a_{\textrm{max}}(t) > t$ is an increasing function of time. The steady state form of $n(a,t\rightarrow \infty )$ is given by the time-independent term in Eq. (5).

## Ensemble Kalman filter

In the first part of this section we describe the basic definitions and update rules in the EnKF [7]. We use the standard state-space representation of a physical system and distinguish between state, output, and input (i.e., control) variables. Outputs are quantities that can be observed or measured (e.g., the number of overdose deaths), while other quantities such as age-specific mortality rates and the number of individuals suffering from SUD are state variables that are not known and have to be estimated. As a first application example, we use the EnKF to estimate the rates $\mu$ and $\lambda$ that arise in Eqs. (4) and (5) of the simple model presented in Sect. 2.

### Basic definitions

To outline the main steps associated with the application of an EnKF to the age-structured partial differential equation (PDE) model in Eq. (1), we primarily follow the notation of Refs. [8, 9]; the EnKF implementation that we use in this work is instead based on Ref. [34].

The evolution of the system state ${\varvec{x}}(t)$ and observed state ${\varvec{z}}(t)$ is described by

7 $$\begin{aligned} \begin{aligned} \dot{{\varvec{x}}}&={\varvec{f}}({\varvec{x}},t)+{\varvec{w}}(t)\quad {\varvec{w}}(t)\sim {\mathcal {N}}({\varvec{0}},{\varvec{Q}}(t))\\ {\varvec{z}}&={\varvec{h}}({\varvec{x}},t)+{\varvec{v}}(t)\quad {\varvec{v}}(t)\sim {\mathcal {N}}({\varvec{0}},{\varvec{R}}(t)) \end{aligned}\,, \end{aligned}$$

where ${\varvec{Q}}(t)$ and ${\varvec{R}}(t)$ denote the covariance matrices associated with the Gaussian process noise ${\mathcal {N}}({\varvec{0}},{\varvec{Q}}(t))$ and Gaussian observation noise ${\mathcal {N}}({\varvec{0}},{\varvec{R}}(t))$ at time *t*, respectively. We assume the quantities ${\varvec{Q}}(t)$ and ${\varvec{R}}(t)$ to be known. The function ${\varvec{f}}(\cdot )$ describes the dynamics of the system state ${\varvec{x}}(t)$, while ${\varvec{h}}(\cdot )$ maps ${\varvec{x}}(t)$ to a measurable quantity. Both functions can be nonlinear.

**Fig. 1:**
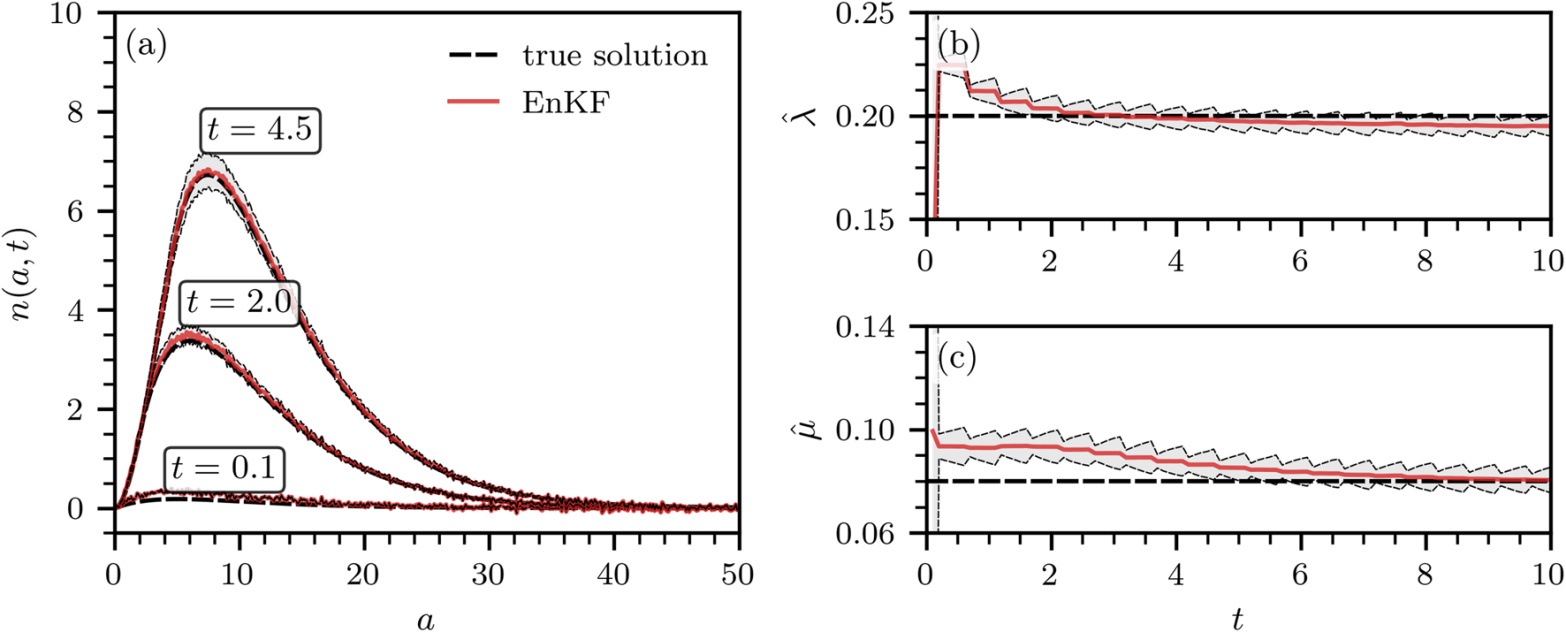
State and parameter estimation with an EnKF. **a** The population with SUD with age between *a* and $a + \textrm{d}a$ at times $t=0.1,2.0,4.5$. **b**, **c** EnKF estimates ${\hat{\lambda }},{\hat{\mu }}$ of the rate parameters $\lambda ,\mu$ [see Eqs. (4) and (5)]. In all panels, the true solution is represented by a dashed black line. Solid red lines and gray-shaded regions indicate EnKF solutions and corresponding $3\sigma$ intervals. The results shown are based on $M=500$ ensemble members

In the context of the age-structured model (1), element $x_j(t)$ of the state vector ${\varvec{x}}(t)$ corresponds to $n(a_j,t)\equiv n(a_0+j\Delta a,t)$ ($0\le j\le {N_a}-1$), the density of individuals whose age lies within the $[a_0+j\Delta a, a_0+(j+1)\Delta a)$ interval at time *t*. That is,

8 $$\begin{aligned} {\varvec{x}}(t)=[n(a_0,t),n(a_1,t),\dots ]^\top . \end{aligned}$$

We use $N_a$ and $\Delta a$ to denote the number of discretizations of the age interval and the corresponding age discretization step, respectively. For the numerical solution of Eq. (7), we later also discretize the simulation time interval [0, *T*] into $N_t$ equidistant intervals of duration $\Delta t=T/N_t$. If we wish to estimate model parameters such as $\mu$ and $\lambda$ introduced in Sect. 2, we can augment the state to obtain

9 $$\begin{aligned} {\varvec{x}}(t)=[n(a_0,t),\dots ,n(a_{{N_a}-1},t),\mu ,\lambda ]^\top . \end{aligned}$$

An example of an inference problem with an augmented state (9) will be provided in Sect. 3.2.

At every time point *t*, the goal of filtering is to determine the state posterior distribution given all prior observations. Before producing EnKF state predictions, we generate an initial ensemble $[\varvec{\chi }_0^{(1)},\dots ,\varvec{\chi }_0^{(M)}]$ that consists of *M* ensemble members $\varvec{\chi }_0^{(i)}\sim {\mathcal {N}}(\hat{{\varvec{x}}}_0,{\varvec{P}}_0)$ ($1\le i \le M$). The quantities $\hat{{\varvec{x}}}_0$ and ${\varvec{P}}_0$ denote the given initial state and covariance estimates, respectively.

We now outline the two main EnKF steps: (i) forecasting the evolution of the system state and (ii) updating the predicted state estimates using observation data. To do so, we discretize the time evolution of the system state and use the shorthand notation $y_k\equiv y(t_k)$ to refer to a quantity *y* at time $t_k=k \Delta t$ ($0\le k\le N_t$). Here and in the remainder of the manuscript, we assume that $t_0=0$.

The basic idea behind forecast and update iterations is that one first uses state estimates $\varvec{\chi }_{k}^{(i)}$ at time $t_k$ to calculate predicted state estimates $\varvec{\chi }_{k+1}^{(i)-}$ at time $t_{k+1}$. These predicted estimates are then combined with observational data to obtain an updated state estimate $\varvec{\chi }_{k+1}^{(i)}$. The superscript “−” in $\varvec{\chi }_{k+1}^{(i)-}$ is used to distinguish the predicted (i.e., prior) state estimates from the updated (i.e., posterior) state estimates.

(i) **Forecast step:** For each ensemble member, we calculate the predicted state estimate $\varvec{\chi }_{k+1}^{(i)-}$ according to
  10 $$\begin{aligned} \varvec{\chi }_{k+1}^{(i)-}=\varvec{\chi }_{k}^{(i)}+\Delta t\,{\varvec{f}}(\varvec{\chi }_{k}^{(i)},t_k)+\varvec{\epsilon }^{(i)}_{k}, \end{aligned}$$
  where $\varvec{\epsilon }^{(i)}_{k}\sim {\mathcal {N}}({\varvec{0}},{\varvec{Q}}_k)$. For the sake of computational efficiency, we avoid discretizations of the partial derivative of *n*(*a*,*t*) with respect to *a* in the EnKF simulations. In all numerical experiments, we first derive closed-form expressions of the rate of change of *n*(*a*,*t*) to compute predictions $\varvec{\chi }_{k+1}^{(i)-}$ according to Eq. (10). The ensemble mean of the predicted state, $\hat{{\varvec{x}}}_{k+1}^-$, and the corresponding covariance matrix, $({\varvec{P}}_{\hat{{\varvec{x}}} \hat{{\varvec{x}}}}^-)_{k+1}$, are given by
  11 $$\begin{aligned}{} & {} \hat{\varvec{x}}_{k+1}^{-}=\frac{1}{M}\sum _{i=1}^{M}{\varvec{\chi }}_{k+1}^{(i)-} \end{aligned}$$
  12 $$\begin{aligned}({\varvec{P}}_{\hat{\varvec{x}}{\hat{\varvec{x}}}}^-)_{k+1}=\frac{1}{M-1}\sum _{i=1}^M\left[ \varvec{\chi }_{k+1}^{(i)-}-\hat{{\varvec{x}}}_{k+1}^-\right]  \left[ \varvec{\chi }_{k+1}^{(i)-}-\hat{{\varvec{x}}}_{k+1}^-\right] ^\top . \end{aligned}$$
  The covariance matrix $({\varvec{P}}_{\hat{{\varvec{x}}}\hat{{\varvec{x}}}}^-)_{k+1}$ is not required in the EnKF iteration, but it can be used to estimate confidence intervals of $\hat{{\varvec{x}}}_{k+1}^-$.
(ii) **Update step:** We begin with deriving the ensemble mean of the predicted observation
  13 $$\begin{aligned} \hat{{\varvec{z}}}_{k+1}^-\equiv \frac{1}{M}\sum _{i=1}^M{\varvec{z}}_{k+1}^{(i)-}=\frac{1}{M}\sum _{i=1}^M{\varvec{h}}(\varvec{\chi }_{k+1}^{(i)-}) \end{aligned}$$
  as well as the corresponding covariances
  14 $$\begin{aligned} ({\varvec{P}}_{\hat{{\varvec{z}}}\hat{{\varvec{z}}}}^-)_{k+1}&=\frac{1}{M-1}\sum _{i=1}^M \left[ {\varvec{h}}(\varvec{\chi }_{k+1}^{(i)-})-\hat{{\varvec{z}}}_{k+1}^- \right] \\& \quad\times \left[ {\varvec{h}}(\varvec{\chi }_{k+1}^{(i)-})- \hat{{\varvec{z}}}_{k+1}^-\right] ^\top + {\varvec{R}}_{k+1}\\ ({\varvec{P}}_{\hat{{\varvec{x}}}\hat{{\varvec{z}}}}^-)_{k+1}&=\frac{1}{M-1}\sum _{i=1}^M \left[ \varvec{\chi }_{k+1}^{(i)-}-\hat{{\varvec{x}}}_{k+1}^-\right] \\&\quad\times\left[ {\varvec{h}}(\varvec{\chi }_{k+1}^{(i)-})-\hat{{\varvec{z}}}_{k+1}^-\right] ^\top \,. \end{aligned} $$
  The Kalman gain is
  15 $$\begin{aligned} {\varvec{K}}_{k+1}=({\varvec{P}}_{\hat{{\varvec{x}}}\hat{{\varvec{z}}}}^-)_{k+1} ({\varvec{P}}_{\hat{{\varvec{z}}}\hat{{\varvec{z}}}}^-)_{k+1}^{-1}. \end{aligned}$$
  For a given observation ${\varvec{z}}_{k+1}$, the state update of ensemble member *i* is
  16 $$\begin{aligned} \varvec{\chi }_{k+1}^{(i)} = \varvec{\chi }_{k+1}^{(i)-}+{\varvec{K}}_{k+1} \left[ {\varvec{z}}_{k+1}+\varvec{\eta }^{(i)}_{k+1} -{\varvec{h}}(\varvec{\chi }_{k+1}^{(i)-})\right] , \end{aligned}$$
  where $\varvec{\eta }^{(i)}_{k+1}\sim {\mathcal {N}}({\varvec{0}},{\varvec{R}}_{k+1})$. Finally, the updated state estimate and the corresponding covariance matrix are given by
  17 $${\begin{aligned} & \hat{{\varvec{x}}}_{k+1}=\frac{1}{M}\sum _{i=1}^M \varvec{\chi }_{k+1}^{(i)}\\ & ({\varvec{P}}_{\hat{{\varvec{x}}}\hat{{\varvec{x}}}})_{k+1} =({\varvec{P}}_{\hat{{\varvec{x}}}\hat{{\varvec{x}}}}^-)_{k+1}\!-\! {\varvec{K}}_{k+1}({\varvec{P}}_{\hat{{\varvec{z}}}\hat{{\varvec{z}}}}^-)_{k+1}{\varvec{K}}_{k+1}^\top \,.  \end{aligned}} $$

### Estimating model parameters

As a first example of estimating model parameters with the help of an EnKF, we focus on the analytically solvable case from Sect. 2 for which closed-form analytical solutions of *n*(*a*,*t*) can be obtained. Our goal is to estimate $\mu$ and $\lambda$ in Eqs. (4) and (5). We thus augment the state (8) by $\mu ,\lambda$ to obtain

18 $$\begin{aligned} {\varvec{x}}(t)=[n(a_0,t),\dots ,n(a_{{N_a}-1},t),\mu ,\lambda ]^\top . \end{aligned}$$

In accordance with Eqs. (4) and (5), the evolution of *n*(*a*,*t*) is described by

19 $$\begin{aligned} \begin{aligned} \frac{\partial n(a,t)}{\partial t}= {\left\{ \begin{array}{ll} (a-t)\, e^{-\lambda (a-t)-\mu t}\quad &{}(a \ge t)\\ 0\quad &{}(a<t) \end{array}\right. } \end{aligned}\,. \end{aligned}$$

The evolution of the first $N_a$ components of the augmented state (18) is described by Eq. (19). We assume that we can observe perturbed versions of *n*(*a*,*t*) but not $\mu ,\lambda$ (i.e., the measurement function is ${\varvec{h}}({\varvec{x}}(t))=[n(a_0,t),\dots ,n(a_{{N_a}-1},t)]^\top$). To avoid sign changes in the estimates of $\mu ,\lambda$ during the EnKF iterations, we apply an exponential transform to render both estimates positive. That is, we first replace $\mu ,\lambda$ with ${\tilde{\mu }},{\tilde{\lambda }}$ in Eq. (18) and then apply the transform $\mu =\exp ({\tilde{\mu }}),\lambda =\exp ({\tilde{\lambda }})$ before carrying out a prediction step according to Eq. (19).

In our simulations, we consider an age interval of [0, 120] years. We set $N_a=1000$ such that $\Delta a=0.12~\textrm{year}$, and we use a timestep of $\Delta t=0.1~\textrm{year}$. Process and observation noise covariances are assumed to be time-independent and given by ${\varvec{Q}}=10^{-4}J_{N_a+2}$ and ${\varvec{R}}=\textrm{diag}(10^{-4},\dots ,10^{-4})$, respectively. Here, $J_n$ denotes the $n\times n$ matrix of ones. Furthermore, we set the initial state and its covariance matrix to $\hat{{\varvec{x}}}_0=[10^{-5},\dots ,10^{-5},10^{-1},10^{-1}]$ and ${{\varvec{P}}_0=\textrm{diag}(0.5,\dots ,0.5,1,1)}$, respectively.

We generate unperturbed observation data from the model using $\mu =0.08/\textrm{year}$ and $\lambda =0.2/\textrm{year}$. The perturbations that we add to *n*(*a*,*t*) are normally distributed with zero mean and variance $10^{-4}$. Our goal is, given the randomized *n*(*a*,*t*), to estimate the underlying $\mu$ and $\lambda$ with an EnKF and verify the degree of accuracy of our estimates compared to the original values. In real-world applications, new observation data may not be available for each prediction. To account for this potential lack of observation data, we perform update steps (i.e., integrate observation data into our predictions) every five prediction periods.

Figure 1a shows the evolution of both the true solution *n*(*a*,*t*) for which $\mu ,\lambda$ are known (dashed black lines) and of the corresponding EnKF estimates that use the augmented state (18). Gray-shaded regions indicate 3$\sigma$ intervals of the EnKF predictions [see Eq. (12)]. We observe that the EnKF produces estimates of $\lambda$ and $\mu$ that are very close to the true solution after $t\gtrsim 2$ years and $t\gtrsim 7$ years, respectively.

## Application to drug overdoses

We now use an EnKF to combine the model in Eq. (1) with corresponding empirical data taken from the CDC WONDER database. Here different causes of death are classified according to the 10th revision of the International Statistical Classification of Diseases and Related Health Problems (ICD-10). We selected ICD-10 codes T40 (poisoning by narcotics and psychodysleptics) and T43.6 (psychostimulants with abuse potential) and all drug-induced deaths, including unintentional death, suicide, homicide, and death by an undetermined cause. We extracted data for the period 1999–2020.

In order to interface drug overdose data with the analytical setup given in Eq. (1), we identify $n(a,t)\,\textrm{d}a$ as the number of people with SUD (w.r.t. any drug) of ages between *a* and $a +\textrm{d}a$ at time *t*. We also associate the influx into the SUD population with an addiction rate of the non-SUD population: $r(a,t)[N(a,t) - n(a,t)]$, where *N*(*a*,*t*) is the overall population density at time *t* from which we subtract *n*(*a*,*t*), the density of people with an existing SUD. Finally, the prefactor *r*(*a*,*t*) represents an age- and time-dependent addiction rate, which might be modeled [35] or estimated from additional data such as surveys. Including these elements, the model in Eq. (1) is adapted to

20 $$\begin{aligned} \begin{aligned} \left[ \frac{\partial }{\partial a} + \frac{\partial }{\partial t} \right] n(a, t) =&- \mu (a, t) n (a, t) \\&+ r(a,t) [N(a,t) - n(a,t)]\,. \end{aligned} \end{aligned}$$

Equation (20) can be recast in the same form as Eq. (1) via

21 $$\begin{aligned} \left[ \frac{\partial }{\partial a} + \frac{\partial }{\partial t} \right] n(a,t) =&- [\mu (a, t) + r(a,t)]n(a, t) \\ \,& + r(a,t) N(a,t).  \end{aligned}$$

Upon comparing Eq. (21) to Eq. (1) we can identify $\mu (a,t) \rightarrow \mu (a,t) + r(a,t)$ and $p(a,t) \rightarrow r(a,t)N(a,t)$, so that the analytical solutions to Eq. (21) can be written through the proper substitutions in Eqs. (2) and (3). Apart from *n*(*a*,*t*), Eq. (21) contains the functions $N(a,t), r(a,t), \mu (a,t)$. Here we introduce some possible forms for them, based on available data and realistic assumptions. We begin with the entire population density *N*(*a*,*t*).

**Fig. 2:**
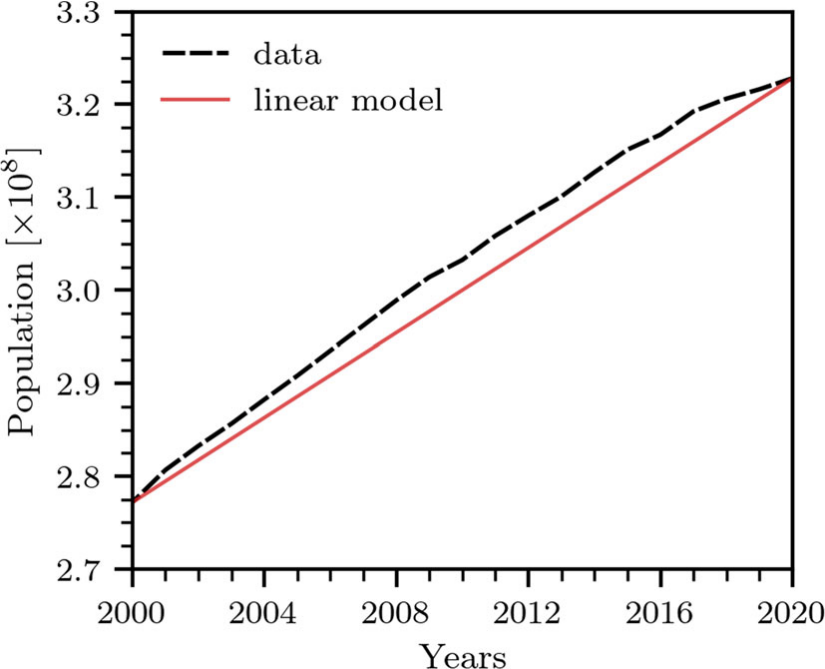
Population growth in the United States between 2000 and 2020. The dashed black and solid red lines show the population growth in the United States during 2000–2020 and a linear population-growth model [see Eq. (22)], respectively

**Fig. 3:**
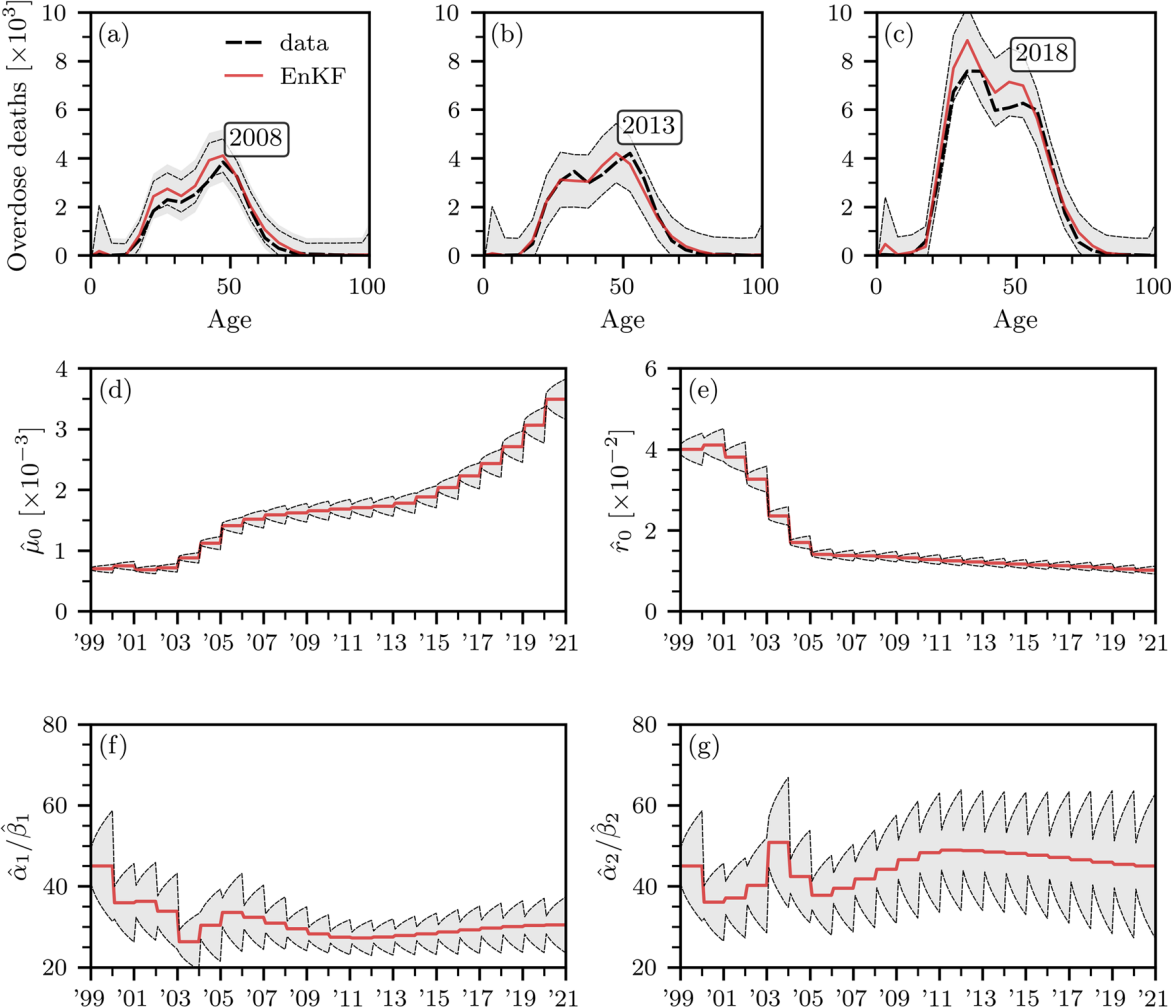
Forecasting overdose mortality and estimating model parameters with an EnKF. **a**–**c** Reported (dashed black lines) and predicted (solid red lines) numbers of overdose deaths in 2008, 2013, and 2018. Empirical data have been collected from the CDC WONDER database. **d**–**g** Evolution of estimated mortality rate ${\hat{\mu }}$, base modulating rate ${\hat{r}}_0$, and gamma function means ${\hat{\alpha }}_1/{\hat{\beta }}_1,{\hat{\alpha }}_2/{\hat{\beta }}_2$. In all panels, solid red lines and gray-shaded regions indicate EnKF solutions and corresponding $3\sigma$ intervals. The shown results are based on $M=10^4$ ensemble members. Observation data for the previous year becomes available in the beginning of every year

Because of different population-level dynamics such as aging and immigration, the population growth in specific age classes is non-monotonic. In principle, it is possible to use interpolation methods and nonlinear functions to construct an age-stratified *N*(*a*,*t*) based on empirical population data that is usually available for 5- or 10-year age windows. However, for the sake of analytical tractability, we will assume an age-independent quantity with *N*(*t*) and focus on the more general form *N*(*a*,*t*) in future work. To account for the quasi-linear US population growth in the past two decades, we set

22 $$\begin{aligned} N(t)=N_0+\Delta N t, \end{aligned}$$

where $N_0=274.9\times 10^6$ and ${\Delta N=2.3\times 10^6}$/year. Figure 2 shows the linear model *N*(*t*) and the corresponding population data for the period 2000–2020.

To model *r*(*a*,*t*), we assume a linear combination of two gamma distributions $f_1(a;\alpha _1,\beta _1),f_2(a;\alpha _2,\beta _2)$, each peaked at different ages, to describe possible variations in the age dependence of the addiction rate. We do this to account for changes in the prevalence of drug type and societal consumption patterns over the 21-year time frame we examine. The quantities $\alpha _1,\beta _1$ and $\alpha _2,\beta _2$ denote shape and rate parameters of the two distributions $f_1$ and $f_2$, respectively. We also assume that *r*(*a*,*t*) does not depend on time and write

23 $$\begin{aligned} r(a,t)\equiv r(a)=\frac{r_0}{2}\left[ f_1(a;\alpha _1,\beta _1)+f_2(a;\alpha _2,\beta _2)\right] , \end{aligned}$$

where $r_0$ is a base modulating rate.

The numerical results that we discuss in the following paragraphs show that a linear combination of two gamma functions allows us to capture the double-peaked distribution of age-stratified overdose deaths [see Fig. 3a–c]. Finally, for analytical tractability of the double integrals arising from the solutions of Eq. (21), we retain the constant mortality rate assumption $\mu (a,t)=\mu$. To combine the mechanistic model in Eq. (21) with empirical data on overdose deaths, we augment the system state ${\varvec{x}}(t)$ [see Eq. (8)] by

24 $$ {\tilde{D}}(a_j,t)=\int _0^t\mu (a_j,t')n(a_j,t')\,\textrm{d}t' \quad(0\le j\le {N_a}-1), $$

where $${\tilde{D}}(a_j,t)$$ is the cumulative number of overdose deaths in the age interval $$[a_j,a_{j+1})$$ up to time *t*. We also augment the system state $${\varvec{x}}(t)$$ by the model parameters $$\mu ,r_0,\alpha _1,\beta _1,\alpha _2,\beta _2$$ that we wish to estimate. As a result, the final augmented system state is

25 $$\begin{aligned} \begin{aligned} {\varvec{x}}(t)=\Big [&n(a_0,t),\dots ,n(a_{{N_a}-1},t),\\&{\tilde{D}}(a_0,t),\dots ,{\tilde{D}}(a_{{N_a}-1},t), \mu ,r_0,\alpha _1,\beta _1,\alpha _2,\beta _2\Big ]^{\top }. \end{aligned} \end{aligned}$$

We derive the corresponding rate of change $\mathrm {\partial }n(a,t)/\mathrm {\partial }t$ for the EnKF updates in Appendix A.

**Fig. 4:**
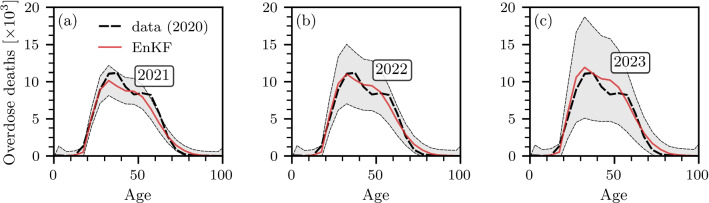
Predicted overdose mortality in 2021, 2022, and 2023. Solid red lines show EnKF predictions of numbers of overdose deaths in **a** 2021, **b** 2022, and **c** 2023. As a reference, dashed black lines show overdose deaths in 2020. Empirical data have been collected from the CDC WONDER database. Gray-shaded regions indicate corresponding $2\sigma$ intervals. The shown results are based on $M=10^4$ ensemble members. The latest observation data that was available to generate the shown predictions is from 2020

For an accurate numerical evaluation of the rate of change of *n*(*a*,*t*), we use a sufficiently small discretization that is associated with age windows that are more granular than the available overdose data. We thus have to coarse-grain the modeled overdose death densities to be able to relate them to observation data. The CDC WONDER data that we use in this work is based on 22 age groups with $a'_0=0,a'_{22}=120$ years and $\Delta a'_1=1,\Delta a'_2=4,\Delta a'_3=5,\dots ,\Delta a'_{21} = 5,\Delta a'_{22}=20$ years. We use a superscript $'$ to distinguish the age discretization in the observation data from the age discretization in the underlying model.

We combine the modeled quantities ${\tilde{D}}(a_j,t)$ with corresponding observation data by numerically integrating ${\tilde{D}}(a_j,t)$ over age windows $[a'_{\ell -1},a'_{\ell })$ ($1\le \ell \le 22$) to obtain the corresponding number of deaths $D(a'_\ell ,t)$ in this age interval at time *t*. Here, $a'_\ell =a'_0+\sum _{m=1}^{\ell }\Delta a_{m}$ for $\ell \ge 1$. Based on the described mapping of ${\tilde{D}}(a_j,t)$ to $D(a'_\ell ,t)$, the measurement function becomes

26 $$\begin{aligned} {\varvec{h}}({\varvec{x}}(t))=[D(a'_1,t),D(a'_2,t),\dots ]^\top . \end{aligned}$$

In our simulations, we set the initial values $n(a_j,0)={\tilde{D}}(a_j,0)=0$. The initial values of $\mu$, $r_0$, $\alpha _1$, $\beta _1$, $\alpha _2$, $\beta _2$ are $7\times 10^{-4}\mathrm {/year}$, $0.04\mathrm {/year}$, 15, $1/(3~\textrm{year})$, 15, and $1/(3~\textrm{year})$, respectively. We have chosen the initial value of $r_0$ in accordance with corresponding empirical data on the number of substance initiates [33]. The initial mean of both gamma distributions is equal to $\alpha _1/\beta _1=\alpha _2/\beta _2=45$ years. To ensure that the parameters $\mu$, $r_0$, $\alpha _1$, $\beta _1$, $\alpha _2$, $\beta _2$ stay positive during EnKF iterations, we use the same exponential transform as in Sect. 3.2. All initial covariances are set to $10^{-4}$, except for the diagonal elements associated with $\mu$, $r_0$, $\alpha _1$, $\beta _1$, $\alpha _2$, $\beta _2$, which are set to $10^{-2}$. The process and observation noise covariances are as in Sect. 3.2. We use a small process noise and a relatively large initial model parameter variance to (i) let the dynamics evolve according to the mechanistic drug overdose model without too much additional noise in *n*(*a*,*t*), ${\tilde{D}}(a,t)$ and (ii) let the filter explore different trajectories associated with appreciable variations in the underlying model parameters. We have also performed simulations for larger process noise values associated with *n*(*a*,*t*) and ${\tilde{D}}(a,t)$. For example, we set the corresponding diagonal elements of *Q* to values between 1 and 100 without observing substantial differences in the simulation results. In the measurement process, we divide ${\tilde{D}}(a,t)$ by $10^3$ to work with numerical values of ${\mathcal {O}}(1)$ when comparing predicted and observed overdose fatalities. A measurement variance of $10^{-4}$ (i.e., a standard deviation of $10^{-2}$) corresponds to about 10–100 overdose fatalities in the simulated data. Although the exact measurement noise is difficult to estimate given the unknown number of undocumented fatal drug overdose cases, we used a standard deviation of about 10–100 as a reasonable modeling choice. In our simulations, we use a relatively large ensemble size of $M=10^4$ to minimize the effect of sampling errors that occur during the Monte Carlo approximation of the system state evolution in the prediction and update steps. Our simulations start in 1998 and we use a timestep of $\Delta t=0.1$ years.

Figure 3a–c shows reported drug overdose deaths (dashed black lines) for the years 2008, 2013, and 2018. Solid red lines represent EnKF predictions that are based on updates that involved observation data from all previous years since 1999. No additional observation data were available between two subsequent years. That is, for predictions that were made for, e.g., 2008, the most recent observation data that was available to the EnKF was from 2007. Still, the EnKF predictions in Fig. 3a–c are closely aligned with the reported overdose deaths. For almost all age classes, predicted overdose fatalities lie within the shown 3$\sigma$ regions (gray shaded regions). For applications in real-time monitoring of overdose fatalities, one may also include provisional data that becomes available during the course of a year to further refine forecasts.

The evolution of ${\hat{\mu }}$ and ${\hat{r}}_0$ [see Fig. 3d, e] suggests that drug overdose mortality increased over the years while the proportion of newly addicted individuals approaches about $0.01\mathrm {/year}$. Up until 2001–2002, the evolution of the mean values of both gamma functions is synchronous [see Fig. 3f, g]. From 2003 onwards, one gamma function captures the addiction dynamics of individuals who are older than 40 years, while the second gamma function captures the inflow of younger people with SUD.

Finally, in Fig. 4, we show forecasts of overdose mortality in the United States for the years 2021, 2022, and 2023. The latest observation data that is available for these forecasts is from 2020 (dashed black lines in Fig. 4). The predicted overdose mortality in 2021 is slightly smaller than in 2020 in age groups between 30 and 60. In 2022 and 2023, the predicted overdose mortality in many age groups exceeds that of 2020. Since the overall overdose mortality has increased unsteadily more than fivefold in the past two decades (with a particularly steep increase between 2019 and 2020), the variance in the shown forecasts is relatively large.

## Discussion and conclusions

We have developed an age-structured model of drug overdose mortality. Our model accounts for age and time-dependent addiction and mortality rates. It can readily be extended to account for multiple drug classes and different ways of stratifying the population. In a simple example, we have shown how age-structured models can be combined with data-assimilation methods such as an EnKF to forecast the evolution of fatalities and estimate model parameters.

Combining our age-specific overdose model with empirical data on overdose fatalities in the United States, we have provided a proof-of-principle set of methods that can be useful for estimating parameters governing drug addiction and mortality and for forecasting the evolution of population-level overdose dynamics.

In addition to developing a framework to include provisional overdose data and retrospective updates of observation data, possible future work includes the study of how regularization terms can help smooth Kalman filter updates [36], or how other ensemble-based Kalman filters, such as ensemble adjustment Kalman filters [37], may help improve numerical stability and forecast accuracy. For applications of the proposed methodology to small population sizes, it might be worthwhile to update the age-stratified population using a Poisson-process model, where the Gaussian noise term only affects the underlying model parameters and not the population numbers themselves. Since we focused on drug overdose forecasting over the past two decades, we decided to use the EnKF in a forward mode and not use backward passes/smoothing (i.e., not use future observations from times $t'>t$ at time *t*). As noted by Evensen and van Leeuwen [38], in forecasting mode, the EnKF and ensemble Kalman smoother (EnKS) produce the same state estimate at the latest time. However, using backward passes and an EnKS (or lagged versions) can help improve earlier parameter estimates, which is also an interesting direction for future research. Another possible direction for future work is to extend the presented data assimilation framework to account for age-dependent death rates $\mu (a,t)$ and age-dependent population data *N*(*a*,*t*) in Eq. (21).

## Data Availability

All mortality datasets are publicly available at https://gitlab.com/ComputationalScience/overdose-da and at https://wonder.cdc.gov/mcd.html.

## Appendix A Rate of change

We evaluate the derivative of Eq. (2) w.r.t. *t* for $t_0=0$, $r(a,t)\equiv r(a)=r_0 \left[ f_1(a;\alpha _1,\beta _1)+f_2(a;\alpha _2,\beta _2)\right] /2$, $N(t)=N_0+\Delta N t$, $\mu (a,t)=\mu$. The resulting rate of change of *n*(*a*,*t*) for $a>t$ is

A1 $$\begin{aligned}  \frac{\partial n(a,t)}{\partial t}=&-\big [\rho ' (a -t)+\rho (a-t)(\mu +r(a-t))\big ]  e^{-\mu t-\int _{0}^t r(a-t+s)\, \textrm{d}s}+r(a)N(t)\\ \,&-\int _0^t e^{-\mu (t-s)-\int _{s}^t r(a-t+s')\, \textrm{d}s'} N(s)\Big (r(a-t+s) \big (\mu +r(a-t+s)\big )+r'(a-t+s)\Big )\,\textrm{d}s.  \end{aligned}$$

For $a<t$, the rate of change is

A2 $$\begin{aligned} \frac{\partial n(a,t)}{\partial t}=\int _{0}^{a} \!r(s)\Delta N e^{-\mu (a-s)-\int _{s}^a r(s')\, \textrm{d}s'}\, \textrm{d}s\,. \end{aligned}$$

The integrals $\int _{s}^t r(a-t+s')\, \textrm{d}s'$, $\int _{0}^t r(a-t+s)\, \textrm{d}s$, and $\int _{s}^a r(s')\, \textrm{d}s'$ can be evaluated using the identity

A3 $$ \int _s^t \frac{\beta ^\alpha }{\Gamma (\alpha )} (a-t+s')^{\alpha -1}e^{-\beta (a-t+s')}\,\textrm{d}s' =\frac{1}{\Gamma (\alpha )}\left[ \Gamma (\alpha ,(a - t +s)\beta )-\Gamma (\alpha ,a\beta )\right] , $$

where $\Gamma (s,x)=\int _x^\infty t^{s-1}e^{-t}\,\textrm{d}t$ denotes the upper incomplete gamma function. We evaluate the remaining integrals $\int _0^t (\cdot ) \,\textrm{d}s$ numerically. The simulation results that we present in Sect. 4 use the age-dependent initial condition

A4 $$\begin{aligned} \rho (a) = 0.04 N_0 f(a;\alpha ,\beta ), \end{aligned}$$

where $f(a;\alpha ,\beta )$ is a gamma distribution with shape and rate parameters $\alpha$ and $\beta$, respectively. In all simulations, we set $\alpha =15$ and $\beta =1/(3~\textrm{year})$ such that the distribution mean is 45 years. The prefactor of 0.04 is chosen such that initially 4% of the population are suffering from a substance use disorder [33].

## Code availability

All source codes are publicly available at https://gitlab.com/ComputationalScience/overdose-da.
